# Protein Expression Platforms and the Challenges of Viral Antigen Production

**DOI:** 10.3390/vaccines12121344

**Published:** 2024-11-28

**Authors:** Jamie R. V. Sookhoo, Zachary Schiffman, Aruna Ambagala, Darwyn Kobasa, Keith Pardee, Shawn Babiuk

**Affiliations:** 1 Canadian Food Inspection Agency, National Centre for Foreign Animal Disease, Winnipeg, MB R3E 3R2, Canada; sookhooj@myumanitoba.ca (J.R.V.S.); aruna.ambagala@inspection.gc.ca (A.A.); 2 Department of Immunology, University of Manitoba, Winnipeg, MB R3E 0T5, Canada; 3 Special Pathogens Program, National Microbiology Laboratory, Public Health Agency of Canada, Winnipeg, MB R3E 3R2, Canada; zachary.schiffman@phac-aspc.gc.ca (Z.S.); darwyn.kobasa@phac-aspc.gc.ca (D.K.); 4 Department of Medical Microbiology, University of Manitoba, Winnipeg, MB R3E 0W2, Canada; 5 Department of Pharmaceutical Sciences, Leslie Dan Faculty of Pharmacy, University of Toronto, Toronto, ON M5S 3M2, Canada; keith.pardee@utoronto.ca; 6 Department of Mechanical and Industrial Engineering, University of Toronto, Toronto, ON M5S 3G8, Canada

**Keywords:** subunit vaccines, protein expression, synthetic biology, viral antigens

## Abstract

Several protein expression platforms exist for a wide variety of biopharmaceutical needs. A substantial proportion of research and development into protein expression platforms and their optimization since the mid-1900s is a result of the production of viral antigens for use in subunit vaccine research. This review discusses the seven most popular forms of expression systems used in the past decade—bacterial, insect, mammalian, yeast, algal, plant and cell-free systems—in terms of advantages, uses and limitations for viral antigen production in the context of subunit vaccine research. Post-translational modifications, immunogenicity, efficacy, complexity, scalability and the cost of production are major points discussed. Examples of licenced and experimental vaccines are included along with images which summarize the processes involved.

## 1. Introduction

Vaccines have been the most successful intervention to respond to infectious disease. There are several types of vaccines including live-attenuated, vectored, DNA or mRNA, killed and subunit. Each vaccine type has its strengths and weaknesses. Although live-attenuated and vectored vaccines can induce strong protective immunity, it is more difficult to demonstrate the safety of these vaccine types, impeding their development as a rapid response to emerging disease outbreaks. DNA or mRNA vaccines use the host cells to produce the antigen offering improved safety over live and vectored vaccines. Killed and subunit vaccines are safer compared to live-attenuated and vectored vaccines, with subunit vaccines considered the most safe [1,2,3,4]. Unfortunately, both killed and subunit vaccines require adjuvants to induce protective immune responses. The immunogenicity of subunit vaccines can be increased by producing virus-like particles (VLPs), which are generated using the component proteins of a virus which spontaneously self-assemble into particles in the absence of a viral genome [5]. The repetitive structure of VLPs stimulates the innate and adaptive immune responses, resulting in improved antibody responses [5,6]. An advantage of subunit vaccines, compared to conventional live-attenuated and most killed vaccines, is their potential for differentiating from infected and vaccinated animals (DIVA) when combined with companion diagnostics [7,8].

The biotechnology industry has rapidly advanced in the past half-century and the expression of recombinant proteins in different systems for a wide variety of applications is a major use of this technology. This has resulted in a range of proteins expressed for therapeutics and diagnostic kits which are discussed elsewhere [2,9,10]. These major advances in genetic engineering have also allowed for the development of recombinant subunit vaccines consisting of protective viral antigens which is the focus of this review. Successful licenced recombinant subunit vaccines have been produced in different protein expression systems such as the Hepatitis B surface antigen (HBsAg) in yeast [3], the human papilloma virus-like particle (VLP) vaccine in baculovirus [4] and several systems for influenza [11,12] and the dengue [13] in mammalian expression systems.

Advances in genetic engineering, including gene synthesis combined with the biotechnology of cell culture systems, has led to the availability of multiple different protein expression systems used to produce recombinant subunit vaccines [10]. After decades of research into the utilities of various protein expression systems, including bacterial, insect, mammalian, plant and cell-free systems, an empirical approach is still required for identifying an appropriate expression system for a specific viral glycoprotein [2,14]. Viral proteins as vaccine antigens often contain post-translational modifications including disulphide bonds and the unique glycosylation patterns that are required for proper folding and/or biological activity, preventing their expression in prokaryotes. The immunogenicity of a recombinant glycoprotein antigen can be highly dependent on its glycosylation and is influenced by the biology of the cell used and the protein being expressed [2,15,16]. Eukaryotic expression systems can be used to express these proteins, including yeast, e.g., *Pichia pastoris* and *Saccharomyces cerevisiae*; baculovirus expression vector systems (BEVSs) using *Autographa californica* multiple nuclear polyhedrosis virus (AcMNPV) and insect cell hosts *Spodoptera frugiperda* (Sf) or *Trichoplusia ni* (Tni); and mammalian cell systems including a variety of transformed cell lines, such as the Chinese hamster ovary (CHO) and human embryonic kidney (HEK)293 [17]. Although all these expression systems can express protein antigens, the glycosylation patterns will differ between the antigen expression systems. The effect of the glycosylation patterns from the different expression systems can be important or not depending on the specific protein use of the protein; this makes the selection of the optimal protein production system difficult without prior knowledge of expression in different systems.

Variations in glycan types and the glycosylation of proteins is a major limiting factor in recombinant antigen production as glycosylation can dramatically impact efficacy. Several glycoproteins carry glycans derived from the initiation of N-Acetylgalactosamine (GalNAc) attached to Ser or Thr residues. Mucins are a class of glycoproteins that have the highest amount of O-GalNAc glycans. These have four core structures which can be extended by sugar residues to produce either linear or branched chains, similar to what is seen on N-glycans. N-glycans are attached by covalent bonds to asparagine residues by N-glycosidic bonds. The synthesis of N-glycans is highly complex in mammalian glycoproteins. Eukaryotic N-glycans have a common core sequence and are then classified into three types: (1) oligomannose, where only mannose residues extend the core; (2) complex types, where ‘antennae’ structures derived from N-acetylglucosamine extend the core; and (3) hybrid types, where mannose extends an arm of the core and one or two N-acetylglucosamine antennae extend another arm [18].

Most viral vaccines produced by recombinant protein expression systems are viral proteins expressed on the surface [19,20,21,22], either capsid proteins in non-enveloped viruses or membrane proteins in enveloped viruses [23,24,25,26,27,28,29,30]. In terms of viral subunit vaccines, *Escherichia coli* (*E. coli*) expression systems have been attempted but have not demonstrated significant enough effectiveness to be commercially viable, though they have been highly effective for bacterial subunit vaccines [27]. Prokaryotic systems have limitations when expressing mammalian (viral) membrane proteins as post-translational modifications are required for their correct folding into a fully functional protein.

This review will discuss the different protein expression systems and their applications for viral antigen expression. In addition, future areas of research for improving protein expression systems focussing on cell-free systems and transgenic animals will be discussed.

## 2. Bacterial Expression Systems

**Figure 1:**
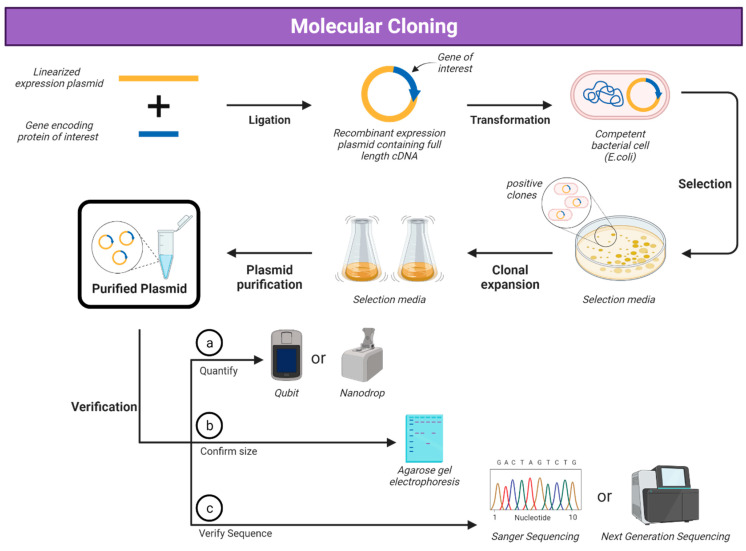
Molecular cloning overview.

**Figure 2:**
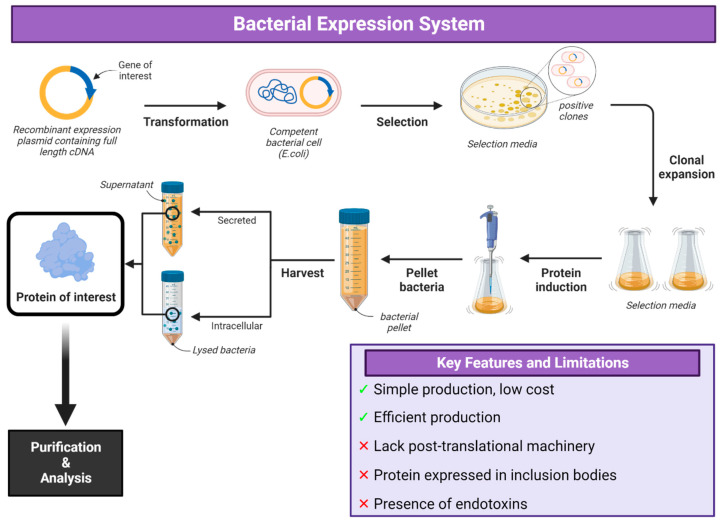
Bacterial expression system.

*E. coli* is the most commonly used bacterial system for protein expression due to its simplicity in being able to use a plasmid encoding the gene of interest to transform the bacteria and the simple production and efficiency of producing protein (Figure 1 and Figure 2) [31,32,33,34,35]. The majority of viral glycoproteins are large proteins (greater than 60 kDa) that are difficult to express using *E. coli.* Though it has been shown that *E. coli* strains such as SHuffle [32] and other strains [33] can produce disulphide bonds in expressed proteins, a lack of post-translational machinery and protein expressed in inclusion bodies make *E. coli* expression not suitable for many viral glycoproteins [34].

Additional issues including the presence of endotoxins such as lipopolysaccharides [35] require additional bioprocessing steps [36] though some species of *E. coli* such as BL21 (DE3) are endotoxin-free [37]. Furthermore, even following endotoxin removal, many proteins still contain level of endotoxin able to activate dendritic cells [38], indicating the difficulty in removing endotoxin from recombinant proteins. In addition, protein expression in bacterial expression systems produces proteins which aggregate in insoluble inclusion bodies, which requires further protein purification procedures to solubilize the protein from the inclusion bodies [39]. Since certain expressed proteins may be insoluble, unstable or inactive without post-translational modifications, *E. coli* may only be the choice expression system for the production of small recombinant proteins without post-translational modification needs [40,41].

### Applications of Bacterial Expression Systems

Several *E. coli* expressed viral antigen vaccine candidates have been developed and are at different stages of evaluation, as summarized in Table 1. The only licenced vaccine was developed by Xiamen Innovax Biotech Co., Ltd. (Xiamen, China) namely the hepatitis E virus (HEV) capsid polypeptide, HEV 239, from *E. coli*, and was the first VLP-based vaccine (Hecolin) against HEV licenced by the FDA of China in 2011 [42]. Antigen production and product consistency was demonstrated at different scales indicating the manufacturing process was robust and scalable [43].

Foot-and-mouth disease virus (FMDV) VLPs have also been produced in this system, providing protection in multiple species [44,45]. VLPs mimicking viral capsids or chimeric VLPs by grafting epitopes of interest onto a VLP display vector are possible platforms for future vaccines via structure-based molecular design. One study used calcium phosphate-mineralized VLPs (CaP-VLPs) and tested three commercial mucosal adjuvants and found that the induction of local mucosal and systemic immune responses via intranasal immunization was specifically enhanced by the adjuvant: STR products [46]. The anti-influenza A M2e-Hbc vaccine candidate, ACAM-FLU-A was produced by *E. coli* using the recombinant hepatitis B core antigen as a carrier VLP, which is an approach that synergizes the benefits of broadly cross-protecting epitopes with rapid scale-up vaccine manufacture using bacterial expression systems [48]. This vaccine resulted in high seroconversion rates in Phase 1 trials but Phase II efficacy trials were cancelled due to a rapid decline in antibody titres [49,50]. Recombinant VLPs for porcine circovirus type 2 capsid protein and porcine parvovirus (PPV) VP2 protein have been expressed in *E. coli* and have demonstrated immunogenicity in piglets and improved growth indices [51].

Since the start of the COVID-19 pandemic, several laboratories have produced SARS-CoV-2 spike and RBD fragments in *E. coli* expression systems [52,53,54,55,56,57]. In these systems, as described above, the proteins are produced insoluble in inclusion bodies which then require extra steps of centrifugation, ultrasonication, redissolving, refolding and then purification. In these studies, only fragments of the SARS-CoV-2 surface glycoproteins approximately 300 bp could be successfully produced. One study [55] was able to produce soluble spike and nucleocapsid protein fragments by fusing the fragments to carrier family 9 carbohydrate-binding module (CBM9) through a flexible linker. Studies using *E. coli* have been successful in producing the RBD of the spike protein [57]. As this system produces little glycosylation, one study [52] showed that non-glycosylated RBD adjuvanted with both 100 μg of Al(OH)_3_ and 50 μg of CpG elicited a robust neutralizing antibody response in mice. While the *E. coli* expression system is a cost-effective system to produce many useful proteins, the requirements for disulphide bond formation and glycosylation for proper expression and folding for viral proteins make this a less desirable system.

## 3. Insect Cell (Baculovirus) Expression Systems

**Figure 3:**
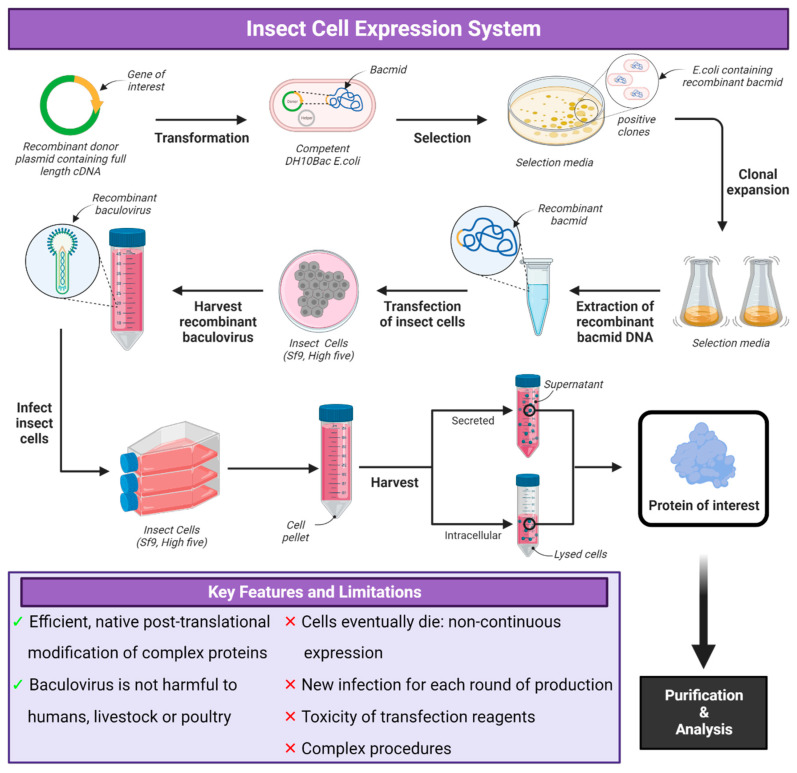
Insect cell (baculovirus) expression system.

Baculoviruses are large, double-stranded DNA viruses which infect insects and have been developed as a recombinant protein expression system using insect cells [58]. To produce recombinant proteins, the baculovirus, *Autographa californica* nuclear polyhedrosis virus (AcNPV), is genetically engineered to encode the gene of the protein of interest. These baculoviruses are then used to infect susceptible insect cells either *Spodoptera frugiperda* (Sf) or *Trichoplusia ni* (Tni) to transiently express the protein (Figure 3).

Homologous recombination is used to generate recombinant baculoviruses in insect cells by the co-transfection of viral DNA and a plasmid containing the gene of interest under the control of the strong polyhedrin promoter of the baculovirus and is flanked by sequences from the polyhedron region [59,60]. The selection of recombinant baculovirus is based on the removal of the polyhedrin gene and the detection of occlusion-negative plaques in insect cell monolayers. Several improvements have been made for the selection of recombinant baculovirus, including the linearization of the virus at the polyhedrin locus [61,62]. Recombination recircularizes the viral genome, which allows for the amplification of the genome [63]. Combinations of this approach were also used, such as the incorporation of a lacZ gene at the polyhedrin locus and the subsequent linearization or engineering of unique restriction enzyme sites into an essential gene (ORF1629) which flanks the polyhedrin locus [64]. The linearization of these sites results in defective gene expression, and the part of the ORF1629 gene that is deleted from the baculovirus genome is incorporated into the transfer plasmid (bacmid) so that ORF1629 function is restored only in the recombinant viruses [65]. The recombinant bacmid DNA is then used to transfect insect cells using a transfection reagent in small cell cultures. Infected cells then give rise to live baculovirions used to infect larger cell cultures to produce and purify the protein of interest expressed in the insect cells [66].

Post-translational modifications are similar, although not identical, to mammalian cells. Glycoproteins produced in insect systems have O- and N-glycans with structures similar to those produced by other eukaryotes, although usually insect expressed proteins do not have antennae of peripheral sugars, but are mainly composed of sialic acids, which are often found on native mammalian glycans [67]. Sialic acids are often found as terminal residues on cell surface glycoproteins and are involved in several immunological interactions in higher eukaryotes. The absence of sialic acids in proteins expressed in insect cells is a significant and somewhat controversial issue in terms of recombinant glycoprotein production. Understanding the important roles that glycan moieties play in protein expression and the need for systems that can produce mammalian-type glycoproteins has led to an increased interest in protein glycosylation pathways and host cells that can be used for functional recombinant glycoprotein expression [68].

Glycoforms of recombinant proteins produced in the BEVS are sometimes different from those produced in mammalian expression systems [69]. Insect-expressed proteins mainly display paucimannose and hybrid-type N-glycans with, at most, minor fractions of complex-type and oligomannose-type N-glycan. Tni cells compared to Sf9 cells can also produce core α-1,3-fucose-linked glycans [70], which may cause hypersensitivity reactions when applied to patients with allergy and therefore should be avoided in cell line engineering [71]. This limitation has been addressed by genetically transforming insect cell lines with constitutively expressible mammalian genes which yield transgenic insect cell lines that can produce humanized recombinant glycoproteins [72]. Genetically modified insect cells include Mimic SF9, SfSWT-1, SfSWT-3, SfSWT-4, SfSWT-5, SfB4GalT, SfRVN^lec1^, DpN1 and A7S [68].

The baculovirus expression system is able to produce complex antigens unable to be effectively produced in prokaryotic systems [73]. However, their use in expressing secreted mammalian proteins is limited for several reasons, the simplest being that insect cell culture media is often just as costly as mammalian cell media. Both insect and mammalian cell lines can be cultured in classical media containing serum, chemically defined media or serum-free media and the overall costs of these are nearly identical for both cell types. Mammalian cells produce glycosylation patterns on proteins that are more representative of those seen in native organisms because insect cells do not produce sialylated complex glycans; however, protein yields in both are similar [73,74]. The construction of the plasmid, amplification, titration and optimization are all time-consuming steps requiring approximately one month or more. Upon infection, the baculovirus genome including the trans-expression cassette is significantly amplified while host synthesis stops. Despite having to compete with viral genes, the expression cassette under the control of the strong viral polyhedrin promoter is itself transcribed and translated at exceptionally high levels. Complex secreted proteins such as viral glycoproteins require several host factors, including glycosylation machinery, chaperones for accurate folding and disulphide isomerases. Under these conditions, the insect cell host machinery is often overwhelmed and so secreted proteins may fail to substantially fold and are instead sometimes found trapped inside the cell in aggregates [75]. Extreme over-expression in the BEVS can result in lower yields of properly folded and secreted protein. This issue has been significantly improved, however, by the inclusion of honeybee melittin, silkworm SP1 and *Drosophila* Bip signal peptides in the trans-expression plasmid, which have allowed for improved and efficient recombinant protein secretion in the BEVS [76,77].

There are several studies which have produced protein antigens in insect cells without the use of the BEVS [77,78,79,80,81,82,83,84,85]. This involves the use of polyethylenimine (PEI)-based transient gene expression and has been shown in both Tni and Sf9 cells. PEI transfection is a high-efficiency nucleic acid delivery system based on transferrin receptor-mediated endocytosis to carry DNA into cells [84]. The expression vector is efficiently delivered by PEI using a variety of different expression vectors and protocols. A higher secreted protein yield has been demonstrated compared to BEVS [79,80].

### Applications of Baculovirus Expression System

There are currently seven licenced BEVS-expressed subunits or VLP vaccines against human and animal viruses (Table 2) [86,87], and several other BEVS viral vaccines in development are described in Table 3. Examples of Drosophila S2 cell line-expressed vaccines are also included [88,89].

Two separate studies indicated the effectiveness of BEVS expressed VLPs produced against chikungunya virus (CHKV). One study showed that a single immunization with 1µg of non-adjuvanted CHIKV-S27 structural polyprotein (C, E3, E2, 6K, E1) induced high-titre neutralizing antibody responses and provided complete protection against viremia and joint inflammation upon challenge using an adult wild-type mouse model of CHIKV disease with the Réunion Island CHIKV strain [96]. The other compared BEVS expressed CHKV VLPs to BEVS expressed CHKV glycoproteins E1 and E2 subunits and showed that the VLPs provided a superior immune response and protection against CHKV-induced disease in mice [97].

A classical swine fever (CSF) BEVS subunit vaccine developed in Vietnam was shown to be safe to administer and effectively stimulated protective immunity against CSFV with a two-dose vaccination [98]. The subunit VN91-E2 recombinant vaccine developed from the E2 CSFV envelope glycoprotein plus complete/incomplete adjuvant was shown to protect experimental pigs against high virulence CSFV’s circulating in Vietnam, including genotypes 1.1, 1.2 and 2.2. E2-specific neutralizing antibodies appeared at day 24, peaked at day 35 and continued until day 63, but transplacental transmission was not described.

Intradermal immunization with VLPs of Ebolavirus (EBOV) GP and VP40 in guinea pigs were shown to induce broad antibody responses and all vaccinated guinea pigs survived challenge with a high dose of guinea pig-adapted EBOV, while all control animals succumbed to the challenge. Both IgG1 and IgG2 antibody responses were elicited by intradermal vaccination with BEVS-expressed EBOV VLPs representing a successful and possibly advantageous route of vaccine against EBOV infection [99].

BEVS recombinant VP2 proteins of epizootic hemorrhagic disease virus (EHDV)-2 and EHDV-6 were used as subunit vaccines in white-tailed deer against the disease. Following vaccination, no deer developed clinical disease or EHDV-induced lesions and in addition, no viral RNA was detected in blood or tissues [100].

Pandemic influenza H1N1 proteins HA, NA and M1 were produced as VLPs using the BEVS and were demonstrated to induce immunity and protect pigs from pandemic influenza challenge. The vaccinations induced robust levels of serum IgG, mucosal IgA and viral neutralizing antibodies. Following challenge with pandemic H1N1 2009, pigs showed reduced lung lesions, viral shedding and the inhibition of viral replication in the lungs compared to unvaccinated pigs [101].

Drosophila S2 cells were used to express recombinant Lassa virus (LASV) glycoprotein (GP) elicited high antibody titres when formulated with a suitable adjuvant after two doses [88]. The BEVS has been demonstrated to express Rift Valley fever virus (RVFV) Gn and Gc glycoproteins, which elicited strong neutralizing antibody responses in sheep and conferred complete protection in all vaccinated sheep demonstrated by the prevention of viremia and fever and the absence of RVFV-associated histopathological lesions [104,105].

Recently, insect cell lines and the baculovirus expression vector system have been used to produce SARS-CoV-2 proteins and VLPs with success [105,106,107,108,109,110,111,112]. SARS-CoV-2 RBD proteins have been expressed and purified in Tni insect cells in a baculovirus mediated Bac-to-Bac expression system in three separate studies, which showed high neutralizing titres [112] and protective immunity in NHPs [105,106].

The E80 and EDIII domains of Zika virus (ZIKV) were produced using the Drosophila S2 cell line and were shown to efficiently elicit protective immunity in a mouse model. Mice were protected against lethal ZIKV challenge by the passive transfer of either anti-E80 or anti-EDIII sera [89].

## 4. Mammalian Expression Systems

**Figure 4:**
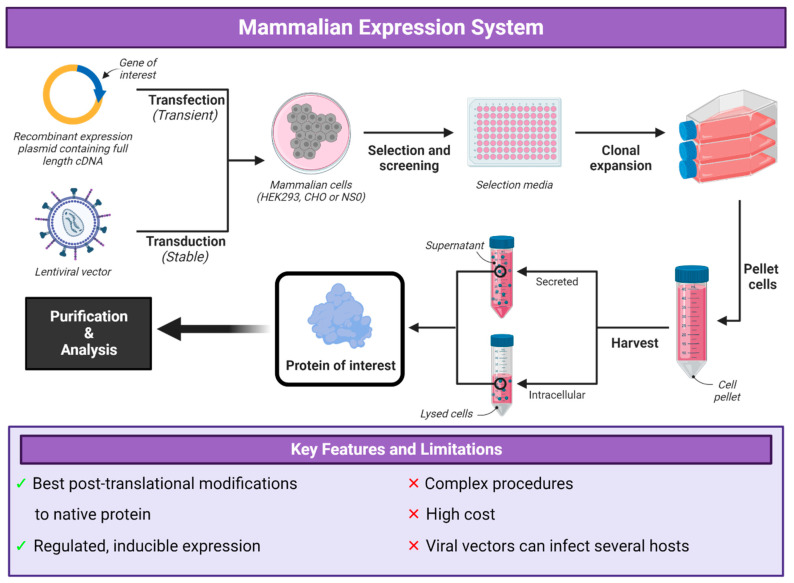
Mammalian expression system.

Mammalian protein expression systems have been modified and optimized over the past twenty years with Chinese hamster ovary (CHO), human embryonic kidney (HEK) and NS0 murine myeloma being the most preferred cell lines (Figure 4) [113]. The advantage to using mammalian cell lines is the requirement for post-translational modifications and the biosynthetic complexity of the proteins of interest [114]. Mammalian cells are more likely to express properly folded proteins with native post-translational modifications. Glycosylation patterns of mammalian-expressed secreted proteins are similar with those observed in vivo with minor differences between species of cell hosts [115,116]. For the expression of the protein of interest, this can be achieved by either transient expression using a plasmid or the generation of a stable cell line using lentiviral vectors. Unfortunately, most mammalian expression systems require using serum to support the growth of the cells, which is costly and requires additional safety testing of the serum used.

Lentiviral vectors play a major role in transferring the gene of interest into the mammalian cell line [117] and their broad tropism allows them to infect most mammalian cell types. Lentiviruses allow for stable integration into the host cell genome ensuring long term expression for stable cell line generation. They can infect both dividing and non-dividing cells such as hepatocytes and neurons, which are known to be difficult to infect. They can deliver transgene fragments as large as 10 kb (~350 kDa), which is well within the range of viral vaccine candidates of 100–200 kDa, and great effort has been put into developing recombinant lentiviruses for clinical and research purposes with viral vaccine development being one major use. Precautions are needed to ensure that replication-competent lentiviruses are not accidentally generated via recombination between the delivered and endogenous viral elements in the producer cells [118,119].

Lentiviral delivery systems have evolved over three generations to reduce biosafety risk. The first-generation system is no longer in use due to biosafety risks. The second-generation system splits essential components of the lentiviral system across three plasmids—transfer, packaging and envelope—that are delivered separately for safety. The transfer plasmid encodes for the transgene and contains cis-acting elements essential for the transcription of viral mRNA and genome packaging. The packaging plasmid is provided in trans and only encodes the essential trans-acting genes required for the entry and integration of the viral genome, and the envelope plasmid contains genes encoding for envelope proteins. The third generation of lentiviral vectors almost completely eliminates dangerous lentiviral recombination events by splitting the viral genome into four plasmids: the packaging plasmid containing only the packaging genes (gag and pol), a regulator plasmid containing only the regulatory gene (rev), a plasmid carrying only the envelope gene (env) and a transgene plasmid containing the gene of interest for producing the vaccine subunit protein [119,120]. Although the third generation is significantly safer, its viral yield is typically lower than that of the second-generation system.

As tropism is determined by the glycoproteins on the surface of the virus, the tropism of a lentivirus can be further broadened by replacing the HIV-1 env glycoprotein with the envelope glycoprotein (G) from the vesicular stomatitis virus (VSV-G). This widens the range of cell types the virus can bind to [116,117,118,119]. Lentiviral vectors have a comparatively high capacity for encoding transgenes and can be engineered with improved safety and efficiency parameters for high transduction efficiency, low anti-vector host immunity, low genotoxicity and persistent gene expression [121,122].

Transient gene expression is the temporary expression of genes following transfection with a plasmid using transfection reagents such as polyethylenimine (PEI) [123,124]. The use of suspension-adapted CHO and HEK cell lines, together with optimized commercial media and inexpensive transfection agents, has allowed transient transfection to be used at production scale to hundreds of litres in bioreactors [125]. This approach requires several milligrams of high-quality, purified plasmid DNA produced by *E. coli*, free of endotoxin and other contaminants [123,126].

Regulated, inducible expression in mammalian cells is often desirable, especially when the product is cytotoxic or cytostatic. Inducible expression is achieved using bacterial gene control elements and transactivator proteins, for example, combinations of hybrid viral transactivators, bacterial repressor proteins and simple chemical inducers provide induction ratios of over 1000-fold [127].

Stable mammalian protein expression can be induced when the transgene expression cassette is replicated extra-chromosomally using viral proteins and *cis*-acting elements, or when integrated into the host genome. This is usually expected for large-scale production and control over protein quality and homogeneity. Transient expression experiments require large quantities of consumable reagents such as transfection reagents and plasmid DNA. Protein yield is often dependent on the transfection efficiency, which varies considerably from one experiment or technician to the next. Protein transiently produced from older cells may not be qualitatively equivalent to protein produced in cells from an earlier passage due to the transformed nature of the cell lines used which increases protein heterogeneity and decreasing protein quality [75,128]. Transient expression requires much less time compared to establishing a stable clonal cell line. However, if several transient transfections are required, the cost of repeated transfections will make the time commitment required for the stable cell line production worthwhile [17].

Stable transgene expression can be maintained using either of two strategies: episomal expression or chromosomal integration and expression. Episomal vectors based on viral elements promote autonomous replication and retention in the nucleus and rely on a *cis*-acting viral origin of replication and *trans*-acting virally encoding protein [129]. Transgene amplification copy numbers can vary drastically, which is a factor that makes it less desirable than stable chromosomal integration [129]. Stable chromosomal integration and expression is reliant on the presence of a suitable selection marker for isolating and screening clonal cell lines. Several selection systems have been developed, such as methotrexate/DHFR and glutamine synthetase protein expression systems, as well as conventional selectable drug markers [130,131]. Stable expression is maintained by physically coupling the transgene expression cassette with a dominant selection marker. One issue with this method is that chromosomal integration is usually a random event, which in some cases results in gene silencing. These integration “position effects” are significant therefore clonal screening is necessary to achieve desirable levels of protein expression [17].

The CHO cell line is widely used due to its ability to grow at high densities in suspension cultures and its ease of adaptation to serum free conditions. This adaptability also has drawbacks as each production target requires the selection of clones that exhibit the necessary phenotypic properties. These include cell doubling time and long-term viability under bioprocess conditions to ensure product quality/uniformity. Even when a appropriate CHO clone is identified, phenotypic drift can occur, making working with CHO clones challenging [132]. For protein expression optimization in CHO cells, glycosylation has been shown to be important, since variation in glycosylation patterns affects protein function and stability, and in addition, non-natural glycoforms can be immunogenic [131,132,133,134,135]. This applies strongly to the terminal α-Gal epitope, which has been added to proteins produced in murine cell lines and is capable of inducing unwanted immune responses in humans [136]. It is therefore essential that CHO production clones are monitored to ensure proper glycosylation. The overexpression of appropriate glycosyltransferases can be used to enhance glycan quality by modifying oligosaccharide structures on a recombinant protein. Improvements are either made by increasing the homogeneity of native structures or introducing non-host cell residues to specialize glycan quality and function [2]. Medium supplementation with glucosamine and *N*-acetylmannosamine allowed for the glycosylation control of a recombinant glycoprotein produced in both NS0 and CHO cells [137,138].

### Applications of Mammalian Expression System

Several licenced (Table 4) and experimental (Table 5) vaccines have been developed using the mammalian expression system. 

The CHO cell line been used to produce a novel subunit vaccine of the CSFV-E2 recombinant fusion protein as a mucosal vaccine. A major issue with most subunit vaccines is the inability to stimulate a significant cell-mediated response, but this study was able to show that the CHO-expressed CSFV-E2-FC protein enhanced IgA levels and induced a CSFV-specific T cell immune response and Th1-biased cellular immune response in pigs. Notably, the efficacy of this mucosal subunit vaccine was found to be comparable to the commercial live-attenuated CSFV C-strain vaccine [152].

A Hendra virus (HeV) G glycoprotein subunit vaccine produced using a soluble form of the HeV attachment (G) envelope glycoprotein (sG_HeV_) made in human HeLa [155,161] and human 293F [156] cells demonstrated protective efficacy in African Green Monkeys [154], cats [155] and ferrets [156] and is currently used to protect horses against Hendra virus in Australia [157]. All studies used CpG oligodeoxynucleotide and Alhydrogel^TM^ as adjuvants. Nipah virus (NiV) and HeV are both closely related, zoonotic paramyxoviruses. In African Green Monkeys, vaccination provided complete protection against subsequent NiV infection with no evidence of clinical disease, virus replication or pathology observed in any challenged subjects [154]. Mucosal immunity was generated in cats vaccinated with recombinant sG_HeV_ as all vaccinated cats possessed antigen-specific IgA on the mucosa. Upon oronasal challenge with NiV, all vaccinated cats were protected from disease even though virus was detected on day 21 post challenge in one cat [155].

Since the start of the COVID-19 pandemic, several studies have been conducted using mammalian expression systems to produce SARS-CoV-2 proteins for serological and vaccine research [159,160,161,162,163,164,165,166,167]. The HPLC analysis of RBD produced in HEK-293T cells was highly homogenous with a sharp elution peak in a reverse phase C18 column and was shown to produce soluble and properly folded polypeptides [163]. Another study, also using HEK-293 cells produced S1 and N proteins that were effective to identify sera from patients positive for COVID-19 by ELISA and immunofluorescence assays [168]. The use of engineered ferritin fused to viral immunogens has also been used to produce RBD vaccines against SARS-CoV-2 [159]. Here, the human codon-optimized RBD was fused to the IL-2 signal peptide at the amino terminus and ferritin at the C terminus to generate an RBD-ferritin fusion. These fusion proteins were readily purified from supernatants of transfected HEK-293 cells. Ferrets were then immunized with the RBD-ferritin fusion plus AddaVax adjuvant and challenged with two different doses of SARS-CoV-2. At challenge doses, the RBD-nanoparticle immunized animals showed strong neutralizing antibody responses and rapid viral clearance in nasal washes and lung tissue vs. control animals. Another study involved the use of an HEK-generated, pan-HLA-DR monoclonal antibody fused to SARS-CoV-2 RBD used as a vaccine in ferrets, which elicited robust protection, strong neutralizing antibody responses and viral clearance in ferret nasal washes vs. control animals [160].

## 5. Yeast Expression Systems

**Figure 5:**
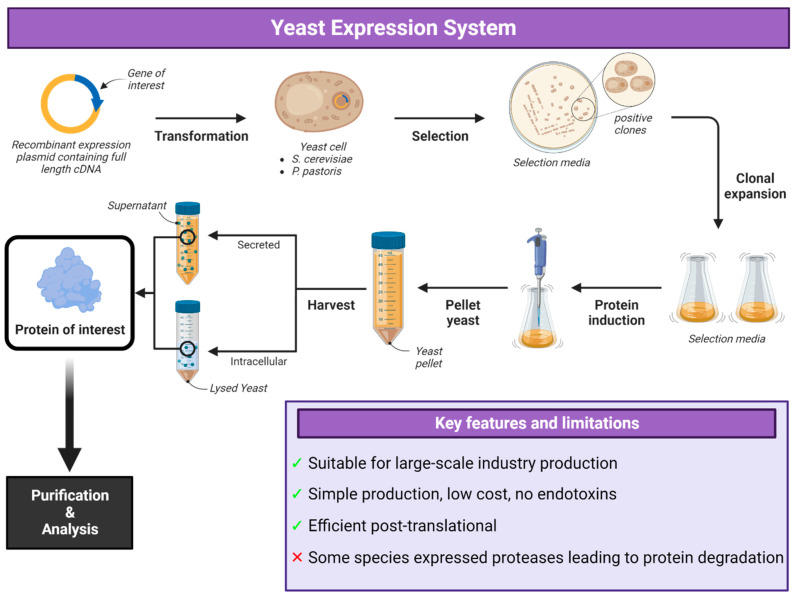
Yeast expression system.

Yeast is the simplest unicellular eukaryotic organism, and the species *Saccharomyces cerevisiae* is just as well studied and characterized as *E. coli*. Hepatitis B virus and HPV recombinant subunit vaccines produced in yeast are the first approved recombinant vaccines for human use [169,170]. The two major systems used for yeast protein expression are *S. cerevisiae* and *Pichia pastoris* (Figure 5) [2]. Yeast systems have been able to produce high yields of secreted recombinant protein while also being fast and relatively inexpensive [171,172]. Culture media for yeast systems can be much less expensive than media for insect or mammalian cell culture, and yeast can easily be cultured to high densities in reusable shaker flasks or at higher densities in oxygen-sparged fermenters. The time commitment for developing a yeast expression cell line is relatively low, cell doubling is rapid and expression experiments are relatively straightforward [173,174]. One well-documented setback is that yeast express several proteases that can lead to significant, but often protein-dependent, protein degradation, though protease deficient cell lines do exist [174,175].

Beyond *S. cerevisiae*, there are other yeast species (*P. pastoris*, *Yarrowia lipolytica* and *Hansenula polymorpha*) that are ‘generally regarded as safe’ (GRAS) for the production of therapeutics and heterologous proteins [176]. Their lower hyper-mannosylation compared to *S. cerevisiae* makes them good candidates for the secretion of human glycoproteins, especially by engineering their glycosylation patterns to resemble human post-translational modification pathways [177]. Both *Pichia* and *Saccharomyces* construct high-mannose-type N-glycosylations on proteins and are incapable of producing more complex glycosylation patterns often needed for efficient bioactivity in mammalian proteins [178]. This may be important, depending on the specific protein being produced. Proteins produced in yeast have a large number of O-glycans and it is usually difficult to predict glycosylation sites. N-glycosylation has been widely studied in yeast, and the signalling pathway has been found to be similar to that in mammalian cells. Glycosyltransferases in mammalian cells are significantly higher than in yeast such as *S. cerevisiae.* Glycosylated proteins in yeast species are also significantly longer than in mammalian cells but the components in mammalian cells are more complex. The engineering of N-glycosylation pathways in several yeast species has allowed for the production of proteins with hybrid N-glycosylation, galactosylation and sialyation complex glycans [179].

Though yeast can provide a robust expression system for many secreted proteins, their divergence from native mammalian post-translational modifications and variability in levels of expression renders them problematic for viral vaccine development, which usually requires the expression of complex mammalian viral surface glycoproteins. Sometimes more than 100 mannose residues are attached, which is called hyper-glycosylation. The artificial in vivo glycoengineering of *P. pastoris* strains has allowed for the development of practical tools for designer glycosylation pathways. This would allow yeast to produce different kinds of glycosylated proteins [180] valuable for recombinant viral vaccine production. Large amounts of protein secreted into an almost protein-free medium makes scaling-up, as well as downstream purification, a much easier task. *Pichia* also does not produce endotoxin, which is a major problem in bacterial expression, and since proteins produced in this yeast system are usually folded correctly and secreted into the medium, the fermentation of genetically engineered *P. pastoris* is an alternative to the *E.coli* system [181]. Yeast cells are also known to be able to enhance expression of MHC and costimulatory molecules on the surface of dendritic cells or antigen presenting cells, which allows for the efficient activation of T lymphocytes promoting cell-mediated immunity [178,179,180]. Though most yeast expression systems produce oligomannose glycans, the polymannose forms of these can be produced and result in strong inflammatory responses in humans. These are sensed by pattern recognition receptors on innate immune cells [182,183,184]. It has been seen that mannans lacking β-1,2-mannoside residues tend to induce the production of higher levels of inflammatory cytokines, such as IL-6 and TNF-α, in mouse DCs [185].

There is an emerging trend toward using *P. pastoris* over *S. cerevisiae* as the choice yeast host species for biopharmaceutical production made evident and as a result of the increased number of patents and publications using *P. pastoris* (see Applications). Host improvements via synthetic biology applications for improved promoters and secretion signals, the development of protease-deficient strains and the humanization of glycosylation patterns can further enhance the recombinant vaccine production potential of several yeast species and has been well-documented in *P. pastoris*. A further advantage includes an increasing toolkit, including the in silico mathematical models of metabolism and genomic approaches for the identification of systems regulating protein production that support the use of *P.pastoris* for biopharmaceutical production [186].

### Applications of Yeast Expression System

The best-known yeast-expressed vaccine is the licenced quadrivalent HPV vaccine Gardasil, which contains VLPs of hrHPV16 and 18 and lrHPV6 and 11, produced in yeast with aluminum hydroxyphosphate sulphate as an adjuvant [187]. Some of the more recent antigens expressed by various species of yeast are shown in Table 6.

Hepatitis C virus envelope glycoproteins E1E2, in both *S. cerevisiae* and *H. polymorpha* as vaccine candidates, have also been included in pending patents. The *H.* polymorpha system produced E1E2 proteins, which were not hyperglycosylated (as the *S. cerevisiae* was) and had sugar chains with a length similar to those produced in mammalian cells. A *H. polymorpha*-derived HCV-envelope protein vaccine were able to clear HCV infection in chimpanzees following challenge [220].

The *P. pastoris* system has been used to produce chikungunya VLPs (CHIK-VLPs), which were evaluated both in vivo and in vitro for efficacy as vaccine candidates. The VLPs elicited high titres of antibodies that recognized native CHIKV and neutralized different strains of CHIKV. *P. pastoris* is in multiple patent filings for expression of flavivirus VLPs proteins as vaccine candidates [221]. The RBD of SARS-CoV-2 has been produced in *P. pastoris* in several studies with appropriate N- and O-glycosylation [163,207,222,223,224,225]. The RBD of SARS coronavirus was expressed in *P.pastoris* X-33. When adjuvanted with aluminum hydroxide, it elicited high neutralizing antibody titres and high RBD-specific antibody titres [226]. RBD produced in the *P. pastoris* X-33 system and adjuvanted with 3M-052 alum was shown to induce robust humoural immunity, strong and durable RBD-specific CD8^+^ and TH1-biased CD4^+^ T cell responses and significantly reduced viral load in LRT and URT in rhesus macaques after SARS-CoV-2 challenge [223]. With the recombinant protein fused to *Saccharomyces cerevisiae* α-factor secretion signal, *P. pastoris* expression yielded 10–13 mg/L in cell culture vs. 5 mg/L in an HEK293 cell line [163]. Yeast-derived SARS-CoV-2 RBD is highly glycosylated and, compared to mammalian and insect cell-derived RBD [105,227], yeast-derived RBD has a very distinct glycosylation pattern. In *P. pastoris*-derived proteins, N-glycans are high-mannose type without core fucose [228] and O-glycans are linear chains of four to five α-linked mannose residues [229]. RBD protein contains complex N-glycans and mainly Core-1 O-glycans when produced in CHO cells [230]. From several studies among expression systems discussed so far, the difference in glycosylation between yeast, CHO and insect-produced RBDs seems to not influence their immunogenicity. PichiaPink™ Strain 1 (Invitrogen, Waltham, MA, USA) cells were also used to produce SARS-CoV-2 RBD as both monomers and dimers, which were shown to elicit broadly neutralizing antibodies and long-lasting protective immunity against SARS-CoV-2 in mice [207]. However, these authors deglycosylated their purified RBD proteins before use in their experiments. Deglycosylation was performed under reducing conditions where purified RBD was denatured then digested with peptide-N-glycosidase F or endo-N-acetylglucosaminidase.

Patent filings have been disclosed for *S. cerevisiae* expressed VP1-4 proteins of enterovirus 71 (EV71) VLPs and for Coxsackievirus A16 (CA16) VLPs, both of which cause HFMD [187]. The VLPs of both viruses showed structural similarities to VLPs produced in EV71-or CA16-infected mammalian cells and mice immunized with the respective VLPs elicited strong and specific neutralizing antibodies and cellular responses that protected neonatal mice against lethal EV71 [231] and CA16 [232] challenge. Yeasts have been used for the production of other viral subunit vaccine antigens as well such as hantavirus, HIV1, poliovirus, rabies and influenza, which have been reviewed previously [233].

## 6. Algal Expression Systems

**Figure 6:**
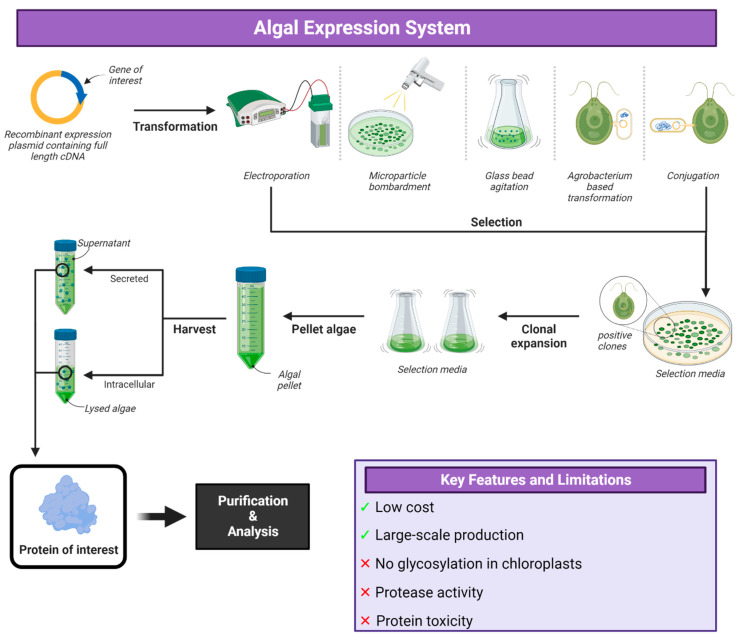
Algal expression system.

Green algae are highly diverse and consist of unicellular and multicellular species. The best characterized and the alga of most interest in vaccine production is the unicellular *Chlamydomonas reinhardtii*. The nucleus, chloroplast and mitochondria of this algal species are easily transformed, and there is a short culture period between generating initial transformants and scale-up to production volumes. Gametogenesis can be induced that allows for genetic crossing; it can be grown either heterotrophically or phototropically, and a wide range of promoters are available and secreted, and glycosylated proteins can be produced and grown in cultures up to 500,000 L in a contained, cost-effective manner (Figure 6) [234,235]. DNA can be delivered via mechanical agitation, surfactant permeabilization, electroporation, particle bombardment or bacterial DNA transfer using conjugation or Agrobacterium-mediated [236,237]. Emerging techniques also exist, such as cell-penetrating peptides, polymers, metal–organic frameworks, nanoparticles and liposomes [238,239,240]. Algae exhibit three times greater photosynthetic efficiency than plants and contain large amounts of protein ranging from 50 to 70% of their fresh weight, which is a much higher percentage than the edible and harvestable parts of any higher plant or animal system [234]. Algae can be lyophilized, and studies have been performed on algal-produced vaccines where dried algae was stored at room temperature for six months [240] and up to twenty months [241]. Protein purified from these stored algae was able to demonstrate antigen effectiveness similar to fresh algae, showing that algae containing unpurified antigen can be stored and transported without the need for a cold chain.

The accumulation of biomass in algae is rapid and the entire biomass can be used for vaccine production, as opposed to plants that expend energy-producing tissues that are not used in the vaccine or cannot be easily harvested. Algae are also not restricted by seasons or soil fertility and media can be recycled to minimize water and nutrient loss. There is no concern regarding the cross-contamination of nearby food crops and enclosed bioreactors can be used to increase biomass yields and reduce concerns of environmental escape [240,242]. While it is evident that algae can produce complex vaccine antigens that are able to elicit immunogenic responses, identifying adjuvants to complement the antigens is critical. Viral antigens in particular require proper post-translational modification, such as glycosylation to be recognized properly by the immune system, but glycosylation does not occur in the chloroplast [243,244,245], so would not be considered suitable for viral vaccine antigen production.

*Dunaliella salina* is another species of algae that has been used to produce recombinant protein. It has a strong adaptability to the environment and can live in various salt concentrations from 0.2% to 35% salt saturation, which prevents contamination from other microorganisms [246]. Like *C. reinhardtii*, *D. salina* is photosynthetic and can be easily, rapidly and inexpensively cultured by large-scale factory farming. As a eukaryotic organism, it can produce and modify recombinant proteins at the levels of transcription and translation. *D. salina* lacks a rigid cell wall and can be easily transformed with exogenous genes [247].

### Applications of Algal Expression System

In recent years, several other algal species have been investigated for their protein expression uses, such as other green algae, diatoms and cyanobacteria, in order to increase host range compatibility [243,244,245]. Over twenty species of algae have been transformed and several promoters and selectable markers have been characterized for many of these species [248,249,250,251].

The RBD of the SARS-CoV-2 spike protein has been produced in algal systems using *Chlamydomonas reinhardtii* [252,253], *Chlorella vulgaris* [254] and *Phaeodactylum tricornutum* [255]. In one study, *Nicotiana benthamiana* agrobacterium-mediated transformation vectors were used to transiently express SARS-CoV-2 RBD in both *Chlamydomonas reinhardtii* and *Chlorella vulgaris* [254] but the biological characterization of the recombinant RBD to the ACE2 receptor as well as the long term expression levels beyond 72 h post-transformation was not investigated. Another study used nuclear transformation in *C. reinhardtii* [252], where stable nuclear genome integration vectors were able to generate recombinant RBD expression levels that were an order of magnitude higher than when using agrobacterium-mediated transformation [254] and maintained this expression for at least one year post transformation. This approach consisted of a nuclear-stable transformation, where a linear vector is randomly integrated in the genome to code a fusion protein RBD::mClover (RBD + a fluorescent protein for monitoring expression). Constructs aimed at inducing expression in the chloroplast and ER, and it was shown that the ER approach was deemed better allowing for yields up to 31 μg/g of fresh algae biomass. The purified RBD from ER-Golgi was also able to bind ACE2 at a similar affinity as mammalian-expressed RBD. It was discussed that higher expression levels using nuclear transformation stems from the use of endogenous promoters, UTRs and a codon-optimized RBD sequence. Using stronger endogenous or synthetic promoters in new transgene designs and the addition of more introns into the RBD coding sequence could be used to boost expression further. However, it is known that several chloroplast genome engineering approaches undertaken in algae that allow for the highest transgene expression also negatively affect the photosynthetic ability of the organism, which removes the option of low-cost photosynthetic cultivation [256]. Another method used is transient expression [253], which implies a short production time and has no need for clone selection and characterization or fusing the antigen to a protein partner, but still yields up to 4 µg/g. Where RBD has also been transiently expressed in *N. benthamiana* [257], yields of up to 8 μg of pure antigen/g leaf mass was recorded. The diatom *Phaeodactylum tricornutum* has an N-linked glycosylation pathway that is like that found in mammals and may be a reason that the RBD made in *P. tricornutum* is bioequivalent to the RBD made in mammalian cells. Expressed RBD in this system was similar to that in *Chlamydomonas* [252]. The RBD purified from whole cell extracts was essentially homogenous and possessed N-linked glycosylation, which suggests that a portion of the expressed RBD is transited through the ER/Golgi apparatus and may be secreted at too low levels to be detected or is not secreted at all. It is possible that other secretory peptides are better optimized for RBD expression and secretion. In purification attempts from *P. tricornutum*, 90–95% of expressed RBD did not bind the Ni-affinity column in their first chromatography step and the majority of RBD was lacking all or some of the C-terminal 6x-His tag, which is possibly because this alga encodes a protease which cleaves the C-terminal 6x-His tag or was due to premature transcription termination, post-translational processing or premature translation termination. Different tags may be better suited for the expression and purification of protein by this algal species.

## 7. Plant Expression Systems

**Figure 7:**
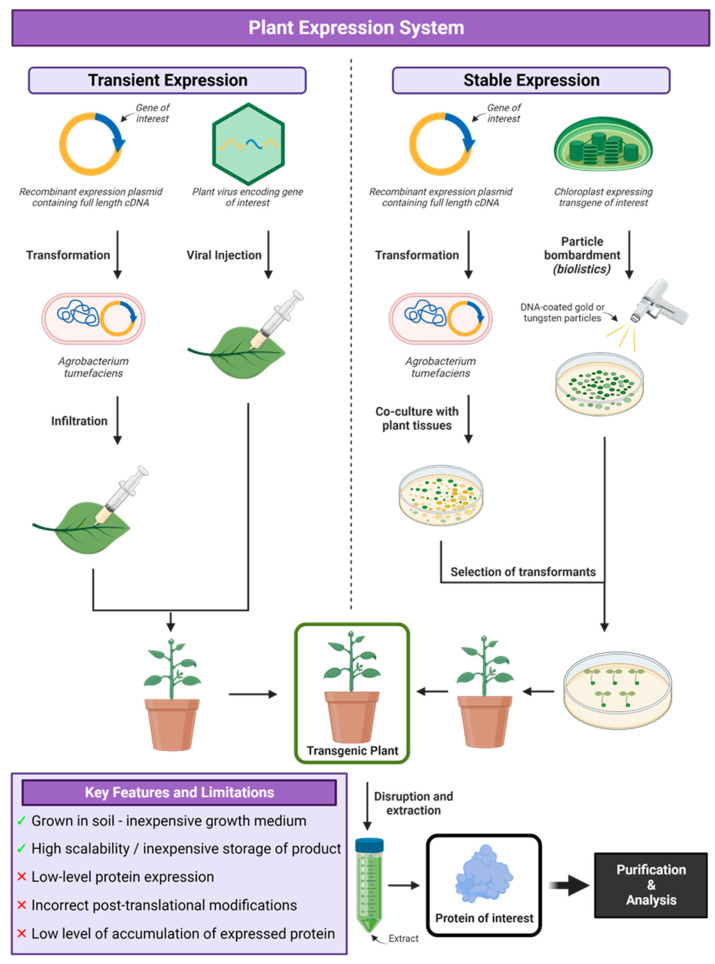
Plant expression system.

Plant-based expression systems are cost-effective, have high scalability and greater safety as plants do not harbour mammalian pathogens (Figure 7). Plants are also able to provide post-translational modifications and appropriate folding resulting in functional, bioactive proteins [258]. Protein production in plants can used either stable or transient expression of transgenes. For stable expression, transgenes can be in the nuclear genome of transgenic plants or plant cell lines or in the chloroplast genome of transplastomic plants. For the transient expression of transgenes in plants an agrobacterial or agroviral vector can be used [258]. Virtually any genetically transformable plant can be used to produce recombinant proteins. Alfalfa, cereal crops like corn, rice and barley, as well as tobacco, are the most widely used as biofactories for biopharmaceuticals [259].

A plant-expressed vaccine should be stable and allow for high expression levels and low production and storage costs. Maize grain has evolved to maintain its proteins in a stable environment to allow germination after years of dormancy, which is in contrast to vegetative matter and fruits which degrade soon after harvesting [260]. This long-term stability has been shown in recombinant proteins where their activity is retained for years stored in the maize grain, which in fact allows for long-term storage and transportation at ambient temperatures and the grain can be processed at will, as opposed to needing to process large batches immediately upon harvest [261]. The removal of oils by supercritical fluid extraction (SFE) can also increase the stability of the grains and the immune response may be enhanced upon SFE treatment [262]. For crop plants, there is a well-established infrastructure for their cultivation, harvest and storage. The protein in cereal grains and oilseeds is stable as these seeds can be stored for long periods of time. Legumes such as alfalfa and soybean can fix nitrogen from the atmosphere and therefore have fewer chemical requirements for growth.

Plant production strategies involve either stable nuclear expression, stable chloroplast expression, transient expression or suspension cultures [263]. Nuclear expression allows for the stable integration of transgenes into the genome of plant cells, which is regulated by the transcription of the gene of interest and then translation in the cytoplasm. *Agrobacterium tumefaciens*-mediated transformation is the most widely used and offers the simplest method for the genetic modification of crops with horizontal transgene transfer and consistent recombinant protein expression. This method, however, can sometimes result in gene silencing, transgene contamination risk, potential interactions with natural products and low yields (<1% of total soluble protein) and can be time consuming [264]. The two methods used involve plant pathogens as vectors, plant viral infection or agroinfiltration by *A. tumefaciens*. High expression efficiency has been observed, but the risk of viral vector contamination and the environment must be carefully considered [265]. Plant cell cultures grown in suspension show more promise than using whole plants and biosafety and environmental concerns can be overcome by using bioreactors to prevent cross-fertilization and the transmission of pollen. Production costs are lower than mammalian systems as they require simple media [266].

The past twenty years has allowed plant expression systems to address technical issues such as expression, yield, purity and reproducibility as well as regulatory expectations like cGMP manufacture and the demonstration of efficacy in pre-clinical and clinical trials. This has allowed plant expression systems to become a commercially appealing approach to develop viral vaccines. In addition to this, there has been a recent shift in the expectation of plants to produce orally administered vaccines to established platforms to produce correctly folded, bioactive proteins, which can be used as injectable vaccines [259]. The major concerns associated with the plant expressed viral/vaccine antigens are oral tolerance, allergenicity, gene transfer to conventional food supplies and potential detrimental environmental impact.

The first USDA-approved plant expressed vaccine was against Newcastle disease in poultry produced by Dow Agro Sciences and was approved in 2006. The vaccine comprises the recombinant haemagglutinin–neuraminidase antigen expressed in transgenic tobacco suspension cells [267,268]. The CSF vaccine, HERBAVAC^®^, was licenced in South Korea. It is a fusion protein of the E2 antigen and the cellulose-binding domain (CBD) and does not contain the E^rns^ antigen present in field strains or live-attenuated vaccine, making it possible for pigs vaccinated with HERBAVAC^®^ to be distinguishable from pigs infected with field strains or vaccinated with the live-attenuated vaccines [269].

### Applications of Plant Expression System

Several plant-expressed viral antigens are in development (Table 7).

H1 and H5 VLP vaccines produced in *Nicotiana benthamiana* elicited both strong antibody responses and poly-functional, cross-reactive memory T cells persisting for at least 6 months post vaccination [270]. Plant-derived VLP vaccines addressed several limitations of currently licenced vaccines. The proteins included in their VLPs are based on the genetic sequence of currently circulating human influenza viruses as opposed to egg-adapted influenza strains for optimal growth. With alum adjuvant, a single intramuscular (IM) injection of 3 μg of a plant-made H7 VLP vaccine induced a robust HI antibody response in both mice and ferrets [271]. The authors were also able to demonstrate clear induction of a strong, antigen-specific, T-cell response after immunization in ferrets. The humoral and cell-mediated immune responses provided 100% protection after lethal challenge with H7N9 in mice and a reduction in clinical signs and virus load ferret nasal and lung washes 3 dpi. Two doses of the unadjuvanted vaccine provided improved protection in mice [271]. This plant-expressed VLP vaccine contains low biological reactivity lipopolysaccharide (LPS) from Agrobacterium, which has been demonstrated to elicit immunological activity and may act as an adjuvant itself [271]. It is possible that this LPS may be partially responsible for the better protection that was observed after two immunizations with the unadjuvanted VLP. An H6 VLP produced in *Nicotiana benthamiana* was used in a challenge study with a heterologous H6N2 influenza virus in chickens [273]. A single dose elicited an immune response comparable to a commercial H6N2 (inactivated) vaccine administered with two doses. The H6 VLP vaccine also significantly reduced the proportion of chicken shedding virus as well as the level of shedding by >100-fold in oral and >6-fold in cloacal swabs.

VLPs displaying an S protein of the infectious bronchitis virus was produced using *Agrobacterium*-mediated *Nicotiana benthamiana* transient expression system [274]. When adjuvanted with Emulsigen^®^-P, specific pathogen-free chickens immunized intramuscularly seroconverted after two weeks with mean HA-inhibition titres of 9.1 to 10 log_2_ depending on the dose. Studies showing virus neutralization potential and/or protection have not been undertaken.

*N. benthamiana* was used to produce HAC1 and HAI-05, recombinant HA antigens of influenza A virus. Both subunits induced significant haemagglutinin inhibition and virus neutralizing serum antibody titres in mice, rabbits and ferrets. The use of Alhydrogel^TM^ adjuvant also provided a dose-sparing effect [272].

A study used pmE2:pFc2 expressed using transgenic plants instead of a transient expression system to produce a CSFV E2 vaccine antigen [276]. While transient expression is generally expected to produce higher amount of target protein than stably expressing transgenic plants [277], the authors used transgenic plants instead as in transient expression systems, the expression level of the target genes is not uniform. Transgenic plants provide the stable expression of recombinant genes once homozygous transgenic lines are generated, which leads to easier large-scale production than in the transient system. Economically, upstream processes in transient expression require additional infrastructure, which can be eliminated in the transgenic approach allowing for inexpensive recombinant vaccine production [278], without lipopolysaccharide contamination [279].

Plant-based manufacturing systems have advantages with scalability compared to tissue-culture and egg-based production platforms and other recombinant technologies with capacity limitations of large-scale bioreactors. In 2012, Medicago demonstrated production of 10 million doses of plant-based H1N1 influenza vaccine within a month, demonstrating the capacity of this system [280,281].

The plant-based production of SARS-CoV-2 antigens has been well studied over the past 3 years, where some of the main drawbacks have been poor reproducibility, antigen stability and bioavailability [275,282,283,284,285,286,287]. A plant expression vector containing SARS-CoV-2 RBD was transformed into *Agrobacterium tumefaciens* strain GV3101 cells by electroporation then injected into 6-week-old *N. benthamiana* leaves [275]. The RBD was then used to immunize mice and NHPs with and without alum and was shown to induce neutralizing antibodies in mice and NHPs. Challenge studies were not performed; however, it was observed that a mixed Th1/Th2 response was induced, and even without alum, a SARS-CoV-2 RBD-specific T cell response was also generated [275].

## 8. Cell-Free Expression Systems

**Figure 8:**
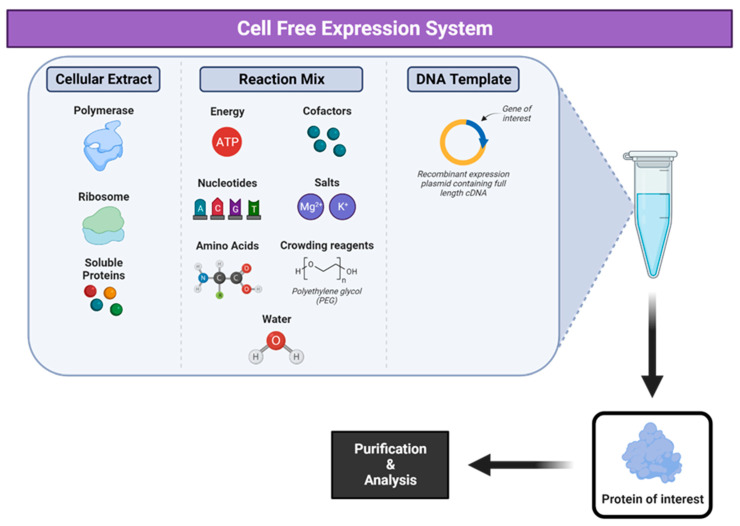
Cell-free expression system.

Cell-free expression systems (Figure 8) (CFSs) replicate the biology of transcription and translation in the absence of a cell. Protein synthesis is initiated in vitro using cell extracts prepared from cells by lysis and several washing steps to remove cell debris and cellular mRNA/DNA [288,289]. The extracts contain the components required for transcription and translation such as aminoacyl-tRNA synthetases, ribosomes, factors necessary for elongation, initiation and transcription. These are combined with amino acids, energy substrates, template DNA, cofactors, salts and nucleotides, and this is varies depending on the biochemical properties of the antigen. Translation is then initiated by adding the linear or circular template DNA or mRNA and the process is allowed to occur at a specified temperature and time period [290].

This work was pioneered in the 1960s [291,292] with major achievements made well into the 2000s, where fundamental ‘operating systems’ were established for proof-of-concept research [293]. The terms ‘cell-free biology’ and ‘cell-free expression systems’ have since evolved to mean much more than described above and now involves the addition of repressor molecules, chaperone proteins, non-native co-factors and substrates to improve the performance and productivity of expression reactions [294,295]. A plasmid containing the gene of interest is directly added to the cell-free expression system to initiate protein production [296]. CFSs can be stored as freeze-dried products allowing for simplified storage and applications [297,298].

Different CFS reaction formats have since been developed, from easily handled and scalable batch reactions with short reaction times but relatively low protein yields to complex dialysis systems known as continuous-flow cell-free and continuous exchange cell-free systems [299,300]. Due to the ease of manipulation of reagents and the scalability of the CFS, automated high-throughput systems are in development [301].

By removing the need to sustain life, CFSs allow control over the molecular machinery for gene expression, enabling the researcher to manipulate the system by adding non-native substrates such as recombinant DNA templates [302]. Cell-free experiments can also circumvent mechanisms involved in species survival and bypass limitations on molecular transport, providing the ability to focus the cellular machinery on the biosynthesis of a specific product. The details of the progress made regarding the preparation of cell-free extracts and reaction mixtures, optimization and more background on cell-free protein expression have been discussed previously [296,297,298,299,300,301]. The most common cell lysates used (Table 8) are acquired from *E. coli* [302,303,304,305,306,307], but other bacterial sources [308,309,310,311,312] or eukaryotic systems such as mammalian [313,314,315,316], insect [299,317,318] or plant cells [319,320,321] have also been adapted, although these are more expensive [322,323].

Prokaryotic CFSs such as *Bacillus subtilis* [310], *Pseudomonas putida* [312], *Streptomyces* [324] and *Vibrio natriegens* [308] have been recently optimized, and eukaryotic CFS based on *Tobacco* [325], *Leishmania* [326], *Neurospora crassa* [327], *Saccharomyces cerevisiae* [328] and erythrocytes [323] have been characterized and optimized for certain proteins at the laboratory level.

Most therapeutic proteins like monoclonal antibodies and viral antigens have glycan moieties. Synthesizing these glycoproteins in vitro is challenging since the most common cellular lysate source for CFSs, *E. coli*, lacks native glycosylation machinery. Several solutions have been suggested such as using a eukaryotic CFS, which can be supplemented with purified microsomes with the necessary components for glycosylation, but these seem to produce much less functional folded protein than bacterial systems [322]. To address these issues, a group developed a novel cell-free glycoprotein synthesis (CFGpS) system which integrates protein biosynthesis with asparagine-linked protein glycosylation. This involved a glycol-optimized *E. coli* strain as the source of cell extracts selectively enriched with glycosylation components such as oligosaccharyltransferases and lipid-linked oligosaccharides. The authors indicated that it enabled a ‘one-pot’ reaction scheme for the efficient and site-specific glycosylation of target proteins [329]. A major advantage of this system is that it allows a high level of control over all glycosylation components (catalysts, substrates and cofactors) in terms of important process variables such as input concentrations, time of addition and reaction time. Genome engineering makes the elimination of inhibitory enzymes, such as glycosidases, possible, which would hydrolyze necessary glycosidic linkages. The level of control with CFSs provides the opportunity to overcome bottlenecks that would otherwise limit glycosylation efficiency [330].

Various methods have been established to enhance the correct folding and solubility of transmembrane proteins such as supplementation with membrane-mimicking structures, such as micelle-forming detergents, nanodiscs, liposomes or exogenous microsomes [324,325,326]. Eukaryotic CFSs contain endogenous microsomes from the cellular endoplasmic reticulum (ER), which allows for the co-translational translocation of proteins and ER-associated post-translational modifications [331,332,333,334,335].

**Table 8:** Comparisons of commonly used CFSs.

System	Advantages	Disadvantages	Reference
*E. coli*	High protein yield. Simple cultivation, rapid cell growth and easy lysate preparation. Cost-efficient. Well-established genetic engineering methods. High levels of VLP production.	Post-translational modification issues. Lack of endogenous membrane structures for integral membrane protein synthesis. Only prokaryotic chaperones. Eukaryotic proteins often incorrectly folded.	[306,307,329,330,331,332,336,337,338,339]
Yeast	Post-translational modifications possible. Rapid, easy propagation of cells and lysate preparation. Well-established genetic engineering methods.	Low protein yield. No mammalian-like post-translational modifications. Slightly more costly than *E. coli*. Moderate levels of VLP production.	[328,333,335,340,341,342]
Wheat germ	High yield of complex proteins. Synthesis of disulphide bridged proteins. Correct protein folding and high solubility. Well-established genetic engineering methods.	Labour-intensive and expensive lysate preparation. Limited post-translational modifications. No endogenous membrane structures. Low protein yield compared to prokaryotic and cell-based wheat germ systems.	[319,334,343]
Tobacco	High yield of complex proteins. Rapid, simple lysate preparation. Glycosylation and disulphide bridge formation possible.	Few studies on tobacco CFSs	[320,325,344]
Insect cell	Rapid, simple lysate preparation. Post-translational modifications possible. Endogenous microsomes available. Direct synthesis and integration of membrane proteins.	High cost of cell propagation. Moderate levels of VLP production.	[299,345,346]
CHO cell	Contains endogenous microsomes. Mammalian PTMs. Direct production of membrane proteins. Well-established cell lines. IRES-mediated translation initiation allows high protein yield.	Low yield compared to prokaryotic CFSs. High cost of cell propagation. Low levels of VLP production.	[347,348]
Human cell	Optimal environment for native protein folding and assembly of viral membrane proteins. Contain endogenous microsomes. Human post-translational modifications. Codon manipulation allows the synthesis of high molecular weight proteins.	Low protein yield compared to prokaryotic CFS. High cell propagation costs. Labour intensive cell culture technologies needed as human cells are sensitive.	[323,349,350]

### Applications of Cell-Free Expression System

The *E. coli*-based CFS has also been used to rapidly produce domains from pH1N1 influenza virus HA. The stabilized HA head domain trimer was effectively recognized by antibodies from pH1N1 influenza vaccine recipients and bound to sialic acids, strongly indicating that the trimers were correctly formed and could be potentially effective as vaccines [351,352]. The *E. coli* CFS has been used to synthesize and assemble human norovirus (HuNoV) VLPs in 4 h with similar protein yield to other cell-based systems [353]. The extremely low level of endotoxin from the cell-free system could meet the demands for vaccine applications, referencing guidelines for toxoid-based vaccines [298,354]. Strains of *E. coli* such as ClearColi^TM^ [37] or the use of an endotoxin binding column are also useful in reducing endotoxin levels.

A wheat germ CFS was used to produce highly soluble MERS-CoV nucleoprotein (NP) antigen, which was able to generate mAbs following the immunization of mice [355]. Hepatitis B core antigen VLPs produced in a *Pichia pastoris* cell-free expression system were found to have comparable characteristics to those previously produced in vivo and in vitro [356].

One study has produced the SARS-CoV-2 disulphide-bonded RBD protein in a wheat germ cell-free production system [357]. A wheat germ (WG) expression kit was used in a bilayer translation method [358], where by adding protein disulfide isomerase (PDI) and endoplasmic reticulum oxidase (ERO1α) to the translational reaction mixture, a functionally intact RBD protein that can interact with ACE2 was synthesized. The common problem with conventional WG expression is the use of dithiothreitol (DTT) in the wheat germ extract and substrate solution to enhance translation efficiency; however, DTT is known to disrupt disulphide bonds in the synthesized protein, which is detrimental to proteins where the disulphide linkage is important to its function. The modification of the WG (EP-WG) with the inclusion of the endoplasmic reticulum oxidoreductase-1 α (ERO1α) and protein disulfide isomerase (PDI) overcomes this drawback to produce biologically similar proteins with intact disulphide bonds. The RBD produced in this system had a different band shape compared to RBD produced in mammalian systems, which is possibly due to glycosylation or other post-translational modifications that occur in the mammalian system. The lack of glycosylation is one of the drawbacks of the WG system. RBD has two glycosylation sites, N331 and N343. In this study, RBD synthesized by EP-WG had slightly lowered affinity for ACE2 than RBD produced in the mammalian cell expression system. Vaccine-derived antibodies produced by conventional WG expression did not react with RBD whose conformation had been disrupted with DTT treatment, demonstrating that the disulfide bonds in the RBD are essential for its antigenicity.

The SARS-CoV-2 RBD has also been produced in a cell-free system derived from *Nicotiana tabacum* BY-2 cell culture (BY-2 lysate; BYL) within 48 h [344]. This work has improved on previous studies [320,325] and has now been optimized and commercialized by LenioBio GmbH as Almost Living Cell-free Extract (ALiCE)^®^. They were able to demonstrate that this system can easily and linearly scale across a 1000-fold difference from 100 μL reactions to 10 and 100 mL. The study also showed preliminary data from 1 L reactions in a commercial bioreactor, the first example of litre scale reactions from a eukaryotic cell-free protein synthesis system. In addition to SARS-CoV-2 RBD, they also produced a hepatitis B core VLP, the monoclonal antibody adalimumab, human epidermal growth factor and a G protein-coupled receptor membrane protein. These expressed proteins are monomeric and multimeric in nature with disulphide bonds, N-glycosylation and transmembrane domains, which demonstrates the versatility of BYL. Using anti-RBD antibodies from R&D systems, serum samples from COVID-19 patients and pure protein samples produced by the BYL-ALiCE cell-free system, RBD-binding and serological assays were performed by ELISA. RBD produced in BYL was directly comparable to RBD produced in HEK-293 cells. The work shows the potential of BYL as an end-to-end R&D protein synthesis platform, which could significantly reduce the time-to-market for vaccine antigens.

## 9. Future Directions

Taking into consideration the time and various complexities involved in traditional cell-based expression, cell-free systems are an emerging vaccine production technology compared in Table 9. Vaccine antigens can be processed for downstream applications like purification and functional analysis without the transfection, selection and expansion of clones. The availability of cell-free lysates from commercial sources has increased; however, there are major challenges with respect to scalability, cost effectiveness, protein folding and functionality.

The BEVS is one of the most efficient cell-based protein production platforms in use as most of the proteins synthesized in this system are functionally, antigenically and immunogenically similar to the native protein. This is because the insect cell system is able to perform several post-translational modifications. Based on the evidence, *Spodoptera frugiperda* CFSs have been developed [366,367]. The ER is not fully removed during lysate preparation but allows the structures to rearrange themselves into endogenous microsomes, which are translocationally active and provide a somewhat natural lipid membrane for protein translocation and embedding. Performing repeated protein synthesis allows the same batch of microsomes to be used multiple times and enrichment of the target proteins in the lumen and membrane of microsomes was achieved [368]. In this insect CFS, post-translational modifications can be achieved without additional enzymes and cofactors as the glycosylation, phosphorylation and disulphide bond formation have been shown [369].

Studies involving CFSs based on CHO cell lysates have shown that the combination of well-known properties and safety aspects of CHO cells [370,371] with the versatility of cell-free technology [314,347,372] provides many advantages for recombinant protein production. The ability to synthesize proteins easily and quickly and the flexibility of the DNA template allows for potential screening technologies. Vaccine antigens are usually membrane proteins originating from humans, and mammalian expression systems fulfil all the requirements such as post-translational modifications, cofactors and chaperones for correct folding and efficient production, although the overexpression of membrane proteins can be toxic for cell cultures and result in cell death or truncated or misfolded proteins [373].

Currently, CHO-lysate CFS reactions are performed coupled with transcription and translation occurring in one reaction, which allows for the fast and convenient production of proteins for high-throughput screening [372]. A major limitation of most eukaryotic CFS is low protein yield, due to limiting translational initiation, but integrating internal ribosome entry sites (IRESs) into the DNA template improved protein production by bypassing several yield-limiting steps in eukaryotic translation initiation [347].

Research into standardization is important as the CFS evolves into a technique used by large laboratories for protein expression and several teams have already begun this work [368,369,370,371,374,375,376,377]. This allows researchers to obtain more statistically significant results and those implementing CFS for the first time can easily and successfully contribute to the establishment of CFS as a major protein expression technology.

Transgenic animals, and more so, transgenic milk, are another recent protein expression platform. Mammary gland-specific promoters have been modified to express a wide range of biopharmaceutical proteins in rodents, rabbits and livestock [378,379,380,381]. The mammary gland is the organ of choice as milk is easily collected in large volumes. Protein expression is commonly reported to be produced at rates of several **grams** per litre [379]. Blood, egg white, seminal plasma and urine are other theoretically possible systems and have been discussed previously [3]. Interest in transgenic eggs has also been shown as a single hen can lay up to 330 eggs per year, and egg white naturally contains approximately 4 g of protein [382]. Urine has also been explored as an abundant biological fluid for protein expression. An advantage of using the bladder is that transgenic animals can urinate earlier than they can lactate; however, the major limitation of this system is yield, as although the bladder epithelium does secrete proteins, production rates are extremely low [383]. Seminal fluid is another relatively abundant biological fluid in some species such as pigs and it can easily be collected. Pig semen contains approximately 30 mg of protein/mL and boars can produce 200–300 mL of semen for a total of 6–9 g of protein per ejaculate [384]. Protein secretion in the male boar’s accessory sex glands is uniquely exocrine, minimizing the risk of a biologically active recombinant protein and upsetting the host’s own physiology; however, it is not well-understood how complex proteins are matured and secreted in semen [385].

In terms of the transgenic animal expression of vaccine antigens, very few studies exist. Enterovirus 71 (EV71) VP1 capsid protein has been expressed and secreted into the milk of transgenic mice with a yield of 2.51 mg/mL. Mouse pups that received VP1-transgenic milk orally showed better health conditions after challenge with EV71 compared to a non-transgenic milk-fed group, and bodyweight of the VP-1-fed group was similar to the uninfected group. Serum neutralization assays and serum antibody detection showed the VP-1-fed mice generated antibodies specific to EV71 [386]. Transgenic rabbits have been used to produce rotavirus VP2 and VP6 proteins in their milk from 50 to 250 µg/mL. These proteins were able to elicit significant protection against rotavirus infection in rabbit babies when orally administered in the whole milk. Protection levels were similar to other studies where VLP2/6 were used as immunogens in oral vaccination protocols.

Recombinant protein production in the milk of livestock seems to be the optimal direction for this type of expression system, which may lead to the creation of animal lines where genomes are modified by the insertion of sequences for the directed recombination of the expression cassette into a milk protein-encoding gene. The insertion can be achieved with high efficiency Via microinjection into the oocytes of a genetic construct carrying an expression cassette as well as the corresponding recombinant integrase or its mRNA. These animal lines can be created using the CRISPR/Cas9 system or its analogues which are highly efficient and simple to design and implement. This is one of the most promising approaches to the generation of transgenic animals producing recombinant antigens in milk as the CRISPR/Cas9 targeted integration of the transgene into the genes that encode milk proteins allows for transgene expression to be controlled by the endogenous regulatory sequences of the recipient gene. Applications of this technology will simplify and standardize transgenic animal production for the generation of recombinant antigens. Synthetic biology allows the use of mini-genes with artificial introns as transgenes to facilitate the efficient expression of the transgene and the production of the target protein, which simplifies the design and creation of genetic constructs for transgenesis [387,388].

The purification of recombinant vaccine antigens from milk creates undefined regulatory hurdles; however, using the modern technology available makes compliance with regulatory requirements describing the transformation event simple. Major hurdles are the requirements regarding the biological safety of recombinant protein in milk, which will require revising the standards for farm animal welfare and veterinary oversight to exclude the presence of extraneous agents as well as controlled parameters of manufactured proteins for use as subunit vaccines.

## 10. Conclusions

Both procaryotic and eucaryotic expression systems have been demonstrated to be useful for production of viral vaccine antigens. However, the number of successfully demonstrated vaccines is much greater in the eucaryotic expressions systems compared to the procaryotic systems. Further advances are underway in improving both procaryotic and eucaryotic protein expression systems to produce proteins with glycosylation patterns like mammalian cells and to be more economical for production. In addition, new cell free systems offer new approaches for protein expression.

## Figures and Tables

**Table 1:** In-development viral vaccines produced using *E. coli* expression.

Virus	Antigen	Findings	Reference
Foot and mouth disease virus	VP0, VP1 and VP3 VLPs	Demonstrated protection in guinea pigs, swine and cattle. Local mucosal and systemic immune responses in mice via IN immunization.	[43,44,45,46]
	VP1	Protected mice and pigs from challenge.	[47]
Influenza A virus	ACAM-FLU-A	Phase I clinical trials.	[48,49,50]
Porcine circovirus type 2	PCV2-Cap VLP	Experimental vaccine demonstrating efficacy in piglets.	[51]
Porcine parvovirus	PPV-VP2 VLP	Demonstrated efficacy in piglets.	[51]
SARS-CoV-2	Dual-adjuvanted RBD	Demonstrated robust neutralizing antibody response in mice.	[52]

**Table 2:** Licenced BEVS-expressed vaccines.

Virus	Vaccine	Antigen	Manufacturer	Reference
Classical swine fever virus	Porcilis^®^ Pesti	E2	MSD Animal Health (Rahway, NJ, USA)	[90]
	BAYOVAC CSF E2^®^	E2	Bayer/Pfizer Animal Health (Leverkusen, Germany)	
Human papillomavirus	Cevarix^®^	L1	GlaxoSmithKline (Mississauga, ON, Canada)	[91]
Influenza A virus	Flublok^®^	HA	Protein Sciences Corporation (Meriden, CT, USA)	[92]
Porcine circovirus type 2	Circumvent^®^ PCV	G2	Merck Animal Health (Rahway, NJ, USA)	[93]
	Ingelvac CircoFLEX^®^	ORF2	Boehringer Ingelheim Vetmedica (Duluth, GA, USA)	[94]
	Porcilis^®^ PCV	ORF2	MSD Animal Health (Rahway, NJ, USA)	[95]

**Table 3:** In-development BEVS-expressed vaccines.

Virus	Antigen	Application	Reference
Chikungunya virus	CHIKV-S27 structural polyprotein (C, E3, E2, 6K, E1)	Experimental vaccine preventing viremia and inflammation in mice	[96]
Chikungunya virus	E1 and E2 proteins	Experimental vaccine demonstrating protection in mice	[97]
Classical swine fever virus	E2 protein	Experimental vaccine demonstrating protective immunity in pigs	[98]
Ebolavirus	GP and VP40 VLPs	Experimental vaccine demonstrating protection in guinea pigs	[99]
Epizootic hemorrhagic disease virus	VP2 protein	Experimental vaccine preventing clinical disease or viremia in deer	[100]
Influenza A virus	HA, NA and M1 VLPs	Experimental vaccine demonstrating protection in pigs against pH1N1 and in chickens against H6N1	[101,102]
Lassa virus	Glycoprotein (GP)	Experimental vaccine eliciting high antibody titres in mice	[88]
Rift Valley fever virus	Gn and Gc Glycoproteins	Experimental vaccine demonstrating complete protection in sheep	[103,104]
SARS-CoV-2	RBD	Experimental vaccine demonstrating protection in NHPs	[105,106]
Zika virus	E80 and EDIII proteins	Experimental vaccine demonstrating protection in mice	[89]

**Table 4:** Licenced mammalian cell-expressed viral antigens.

Virus	Vaccine	Cell Line	Antigen	Manufacturer	Reference
Classical swine fever	Porvac^®^	HEK 293	E2-CD154	The Centre for Genetic Engineering and Biotechnology (CIGB) (Havana, Cuba)	[139]
Dengue virus	Dengvaxia^®^	Vero	preM and E	Sanofi Pasteur (Val de Reuil France)	[13]
Influenza A (H1N1) 2009	Celvapan^®^	Vero	Whole virion	Baxter (Orth an der Donau Austria)	[140,141]
Influenza A (trivalent)	Flucelvax^®^	MDCK	HA and NA	Novartis Vaccines and Diagnostics (Basel, Switzerland)	[12,142]
Influenza A (trivalent)	Preflucel^®^	Vero	HA	Baxter (Orth an der Donau Austria)	[11]
Influenza H5N1	Celvapan^®^	Vero	Whole virion	Baxter (Orth an der Donau Austria)	[140,141]
Respiratory syncytial virus	AREXEVY^®^	CHO	RSVPreF	GlaxoSmithKline (London, UK)	[143,144,145,146,147]
Respiratory syncytial virus	ABRYSVO^®^	CHO	RSVPreF	Pfizer (New York, NY, USA)	[148,149,150,151]

**Table 5:** In-development mammalian cell-expressed viral antigens.

Virus	Antigen	Cell Line	Application	Reference
Classical swine fever virus	E2 Protein	CHO	Experimental mucosal vaccine demonstrating protection in pigs.	[152]
Hendra virus	sG_HeV_	HeLa	Experimental vaccine demonstrating protective efficacy in African green monkeys and cats.	[153,154,155]
Hendra virus	sG_HeV_	293F	Experimental vaccine demonstrating protective efficacy in ferrets and horses.	[156,157,158]
SARS-CoV-2	RBD-nanoparticle	HEK-293	Experimental vaccine eliciting strong neutralizing antibody responses and rapid viral clearance in nasal washes.	[159]
SARS-CoV-2	Pan-HLA-DR mAb fused to RBD	HEK-293	Experimental vaccine demonstrating robust protection, strong neutralizing antibody responses and viral clearance in ferret nasal washes.	[160]

**Table 6:** In-development yeast-expressed viral antigens.

Yeast Species	Virus	Antigen	Reference
*H. polymorpha*	Papillomavirus	L1 protein	[188]
	Hepatitis B virus	VrHB-IB	[189]
	Porcine circovirus type 2	PCV2b capsid protein	[190]
*P. pastoris*	Dengue virus	Envelope glycoproteins	[191,192,193,194,195]
	Hand, foot and mouth disease (HFMD)	P1 and 3CD proteins	[196]
	Hepatitis B virus	HBsAg	[197]
	Hepatitis C virus	HCV Core protein	[198]
	Papillomavirus	HPV 16L1, 18L1	[199,200]
	Influenza virus	Hemagglutinin protein	[201,202,203]
	Classical swine fever virus	E2 glycoprotein	[204,205]
	Chikungunya virus	Structural protein VLPs	[206]
	SARS-CoV-2	RBD monomers and dimers	[207]
*S. cerevisiae*	Papillomavirus	VLPs of hrHPV16 and 18, and lrHPV6 and 11—Gardasil^®^	[187]
		HPV16	[208]
	Hepatitis B virus	Hepatitis B surface antigen (HBsAg)—Recombivax^®^	[209,210]
		HBV X, S and C antigens	[211,212]
		Surface protein GS-4774	[213]
	Enterovirus 71 (hand, foot and mouth disease)	EV71 Structural antigens	[214,215]
	Parvovirus B19	VP	[216,217]
	Dengue virus	Dengue envelope domain III	[218]
	Human immunodeficiency virus-1	Envelope glycoprotein	[219]

**Table 7:** In-development plant-expressed viral antigens.

Plant	Virus	Antigen	Application	Reference
*Nicotiana benthamiana*	Influenza	H1 and H5 VLPs	Experimental vaccine eliciting strong humoral and long-term cell-mediated responses in adults.	[270]
	Influenza	H7N9 VLP	Experimental vaccine demonstrating strong antibody response in mice and ferrets.	[271]
	Influenza	HA subunit	Experimental vaccine demonstrating immunogenicity in mice, rabbits and ferrets.	[272]
	Influenza	H6 VLP	Experimental vaccine demonstrating reduced viral shedding in chickens.	[273]
	Infectious bronchitis virus	S protein VLP	Experimental vaccine demonstrating immunogenicity in chickens.	[274]
	SARS-CoV-2	RBD	Experimental vaccine shown to induce neutralizing antibodies in mice and NHPs.	[275]

**Table 9:** Comparison of cellular vs. cell-free antigen expression systems.

	Cellular Expression	Cell-Free Expression	References
**Time**	1–2 weeks	24–72 h	[308,344]
**Protein Toxicity**	Often a major issue	High tolerance for toxic proteins	[289,359]
**Membrane Proteins**	Overexpression can cause cytotoxicity and cell death	Variety of different sizes can be produced depending on lysate source	[331,332,360]
**Protein Yield**	High yields (mg/mL)	Yield varies depending on the protein from µg/mL to mg/mL	[288,304]
**Post-translational Modifications**	All possible depending on the system.	Mainly in eukaryotic CF systems with translationally active microsomes. Limited in prokaryotic and eukaryotic lysates without endogenous microsomes. O-linked glycosylation does not occur.	[302,361]
**Scalability**	Minimum volume of 5 mL to several litres.	Range from 5 µL (chip-based, Eppendorf tube) to 100 L (fermenter, commercial bioreactor)	[362,363]
**Flexibility and Manipulation**	Closed system, difficult to manipulate	Open system, easy to manipulate reaction conditions, no cell membrane constraints.	[364]
**Point-of-care protein production**	Difficult due to time-consuming process, infrastructural facilities and cold storage.	Lyophilization allows for removal of cold-chain and on-site production without major facilities.	[298,365]
**Applications**	Cells need to be lysed for membrane protein applications.	Simple as protein can be purified and reconstituted immediately post-synthesis.	[288,364]
**Acceptance**	Reliable, current standard for protein production approved by regulatory authorities.	Currently limited to research at laboratory level with few, limited commercial applications	[289,304,344]

## Data Availability

The data and information presented in the review are from papers published (and cited) in the References section, with abstracts accessible in PubMed.
